# Contralateral Recurrences of Post-vaccination Multiple Evanescent White Dot Syndrome

**DOI:** 10.7759/cureus.32300

**Published:** 2022-12-07

**Authors:** Estefania Ramirez Marquez, Sofía C Ayala Rodríguez, Laiza Rivera, Mariella C Pappaterra-Rodriguez, Guillermo A Requejo-Figueroa, Radames Rios, Erick Rivera-Grana, Eduardo J Rodríguez-García, Armando L Oliver

**Affiliations:** 1 Ophthalmology, University of Puerto Rico School of Medicine, Medical Sciences Campus, San Juan, USA; 2 Medicine, Ponce Health Sciences University, Ponce, USA

**Keywords:** case report, brown subretinal lesions, covid-19, meningococcal vaccine, human papilloma virus vaccine, multiple evanescent white dot syndrome

## Abstract

We report on a case of multiple evanescent white dot syndrome (MEWDS) following the simultaneous administration of the human papillomavirus and meningococcal (conjugate) vaccines and two recurrences of MEWDS following the administration of the second dose of the human papillomavirus (HPV) vaccine and the COVID-19 vaccine and COVID-19 viral infection. A 17-year-old Hispanic female presented with a one-week history of photopsia and blurred vision in her left eye following the simultaneous administration of the human papillomavirus and meningococcal (conjugate) vaccines. Upon a comprehensive examination, her best-corrected visual acuity was 20/20 in the right eye and 20/100 in the left eye. A left fundus examination revealed multiple white dots in the macula and nasal periphery, consistent with a diagnosis of MEWDS. Ancillary testing, including fundus autofluorescence, fluorescein angiography, indocyanine green angiography, and optical coherence tomography, supported the diagnosis. One month following her initial diagnosis, the patient's symptoms had resolved without any therapy, and a fundus examination revealed multiple relatively ill-defined brown-colored subretinal lesions in the nasal midperiphery, corresponding to the location of the previous MEWDS lesions. Subsequently, she received the second dose of the HPV vaccine and then developed a mild COVID-19 infection. Four months after the initial presentation, she received the first dose of the BNT162b2 COVID-19 vaccine, followed by the second dose a month later. Eight months following her initial presentation, she presented with photopsia in the right eye. Her visual acuity remained 20/20 in the right eye and improved to 20/20 in the left eye, and white dots were identified nasal to the disk and surrounding the peripapillary region; the contralateral MEWDS diagnosis was confirmed by the previously mentioned ancillary tests. At her one-month follow-up, she presented new onset photopsia of the right eye. Her visual acuity remained 20/20 in both eyes, and a fundus examination revealed white lesions suggestive of active MEWDS temporal to the macula and brown-colored spots nasal to the disk, suggestive of recovering MEWDS, nasally. The aforementioned testing confirmed the coexistence of new and resolving lesions; nonetheless, the patient's symptoms resolved without any therapy, and she received the third dose of the BNT162b2 COVID-19 vaccine 11 months after her initial presentation. Our case suggests that vaccines may serve as immunological triggers of MEWDS. Recurrent MEWDS may occur when an individual is exposed to a powerful immune challenge, such as receiving a wide array of vaccinations in a short period of time. We believe this case constitutes a previously undescribed finding of multiple relatively ill-defined brown-colored subretinal lesions present in late MEWDS.

## Introduction

Multiple evanescent white dot syndrome (MEWDS) is characterized by distinct small, round, white spots scattered over the posterior pole and midperiphery [1-3]. As the name MEWDS intimates, the white dots usually fade and resolve within a short period [1-3]. Patients may report accompanying brief and intermittent episodes of flashes of light (photopsia), which do not tend to require any therapeutic intervention [2,4,5]. This disease has been described in the literature as multifocal retinopathy that involves the retinal pigmented epithelium as well as the outer retina, and it is most commonly found among young women [2,4,5]. Because MEWDS may be accompanied by a flu-like prodrome, viral-mediated infections, vaccines, and autoimmune mechanisms have been proposed as their triggers, but the etiology and pathophysiology of this disease remain mostly uncertain [1-3].

Multiple evanescent white dot syndrome was first described by Jampol et al. in 1984 with two cases that were unilateral and monophasic [2,6]. At the present time, various authors have described cases of bilateral, recurrent MEWDS [2,3]. We herein present the case of a woman with bilateral recurrences of MEWDS that were temporally related to exposure to a wide array of antigens from various vaccines and to her having been infected with COVID-19.

## Case presentation

A 17-year-old Hispanic female presented with a one-week history of photopsia and blurred vision in her left eye (OS). Her past medical history was remarkable for her having received the human papillomavirus (HPV) and meningococcal (conjugate) vaccines simultaneously 16 days before the onset of her symptoms. Her review of systems, as well as past social and family history, were otherwise unremarkable.

**Figure 1:**
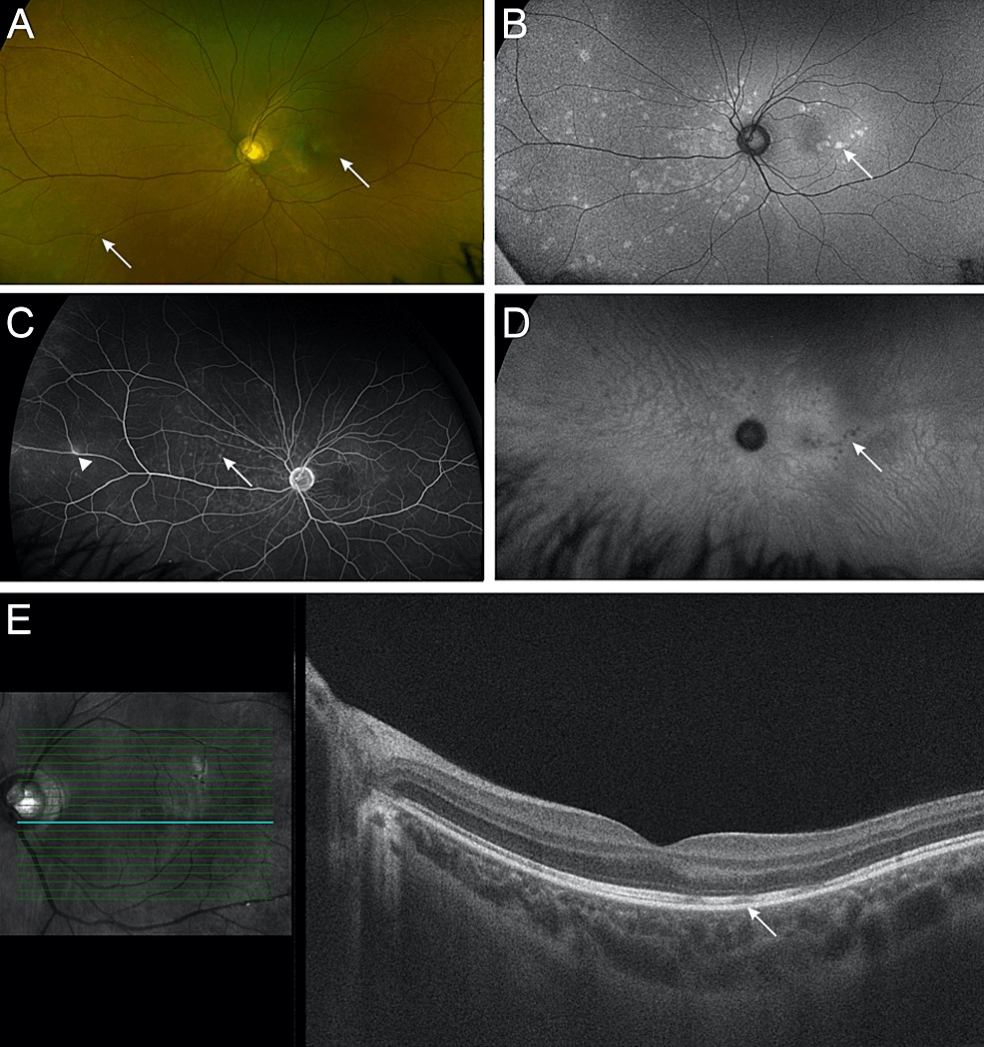
Ultra-widefield fundus imaging and optical coherence tomography of the left eye at presentation (A) Color photographs reveal multiple white dots (arrows) in the macula and nasal periphery. (B) Fundus autofluorescence, marked by hyperfluorescent spots (arrow) in the macula and nasal periphery in a similar pattern as that observed in the fundus examination. (C) A late-phase intravenous fluorescein angiogram shows hyperfluorescent spots (arrow) and mild periphlebitis (arrowhead) in the nasal midperiphery. (D) Late-phase indocyanine green angiography reveals spots (arrow) around the macula and nasal midperiphery. (E) Spectral-domain optical coherence tomography reveals numerous focal hyporeflective lesions (arrow) at the inner segment/outer segment junction, which correlate with the macular lesions.

Upon a comprehensive ophthalmic evaluation, her best-corrected visual acuity (BCVA) was 20/20 in the right eye (OD) and 20/100 OS with a manifest refraction of plano -0.75 x 105 OD and -1.50 -0.50 x 80 OS. The intraocular pressure was 19 mmHg in both eyes (OU). The pupils were round and reactive to light, and there was no afferent pupillary defect (APD). Color vision OU, as assessed by the Ishihara color plate test, revealed no defect. Extraocular movements were within normal limits. A slit-lamp examination was within normal limits, bilaterally, with no evidence of keratic precipitates, signs of inflammation in anterior chambers, or vitreous cells in either eye. The patient's right fundus was unremarkable; however, the left fundus revealed multiple white dots in the macula and nasal periphery (Figure 1A).

The fundus autofluorescence (FAF) was within normal limits in the OD; however, in the OS, it was marked by hyperfluorescent spots in the macula and nasal periphery in a similar pattern as what was observed in the fundus examination (Figure 1B). The intravenous fluorescein angiogram (FA) showed normal findings in the OD; in the OS, it revealed hyperfluorescent spots and mild periphlebitis in the nasal midperiphery (Figure 1C). The patient's indocyanine green angiography was unremarkable within normal limits in the OD; in the OS, it was remarkable for spots around the macula and nasal midperiphery (Figure 1D). A spectral-domain optical coherence tomography (OCT) of the OD was within normal limits; however, the OS revealed numerous focal hyporeflective lesions at the inner segment/outer segment (IS/OS) junction, which correlated with the macular lesions (Figure 1E).

Provided with the clinical and ancillary testing results, an assessment of MEWDS was made, and a workup was ordered to rule out secondary etiologies, such as syphilis and sarcoidosis. All the tests, including fluorescent treponemal antibody absorption, rapid plasma reagin, and a chest x-ray, were negative. The patient was placed under close observation without any topical or systemic therapy.

One month following her initial diagnosis, the patient's symptoms had resolved. The patient's BCVA remained 20/20 OD and improved to 20/20 OS. A fundus examination revealed multiple relatively ill-defined brown-colored subretinal lesions in the nasal midperiphery corresponding to the location where the MEWDS lesions were present. The FAF and FA hyperfluorescent spots in the macula and nasal periphery had resolved. The corresponding OCT images of the OS showed that the numerous focal hyporeflective lesions previously found at the IS/OS junction had been resolved.

The patient received the second dose of the HPV vaccine series two days after her follow-up. She later developed COVID-19 (six weeks after her initial presentation). Her symptoms were mild, including anosmia and nasal congestion; nevertheless, she evidenced no ocular symptoms during the course of the infection. Three months following her initial visit, a color fundus photograph, and FAF revealed that the lesions had resolved; she presented no further complaints at this time. Four months after the initial presentation, the patient received her first dose of the BNT162b2 COVID-19 vaccine, which was followed by the second dose a month later.

**Figure 2:**
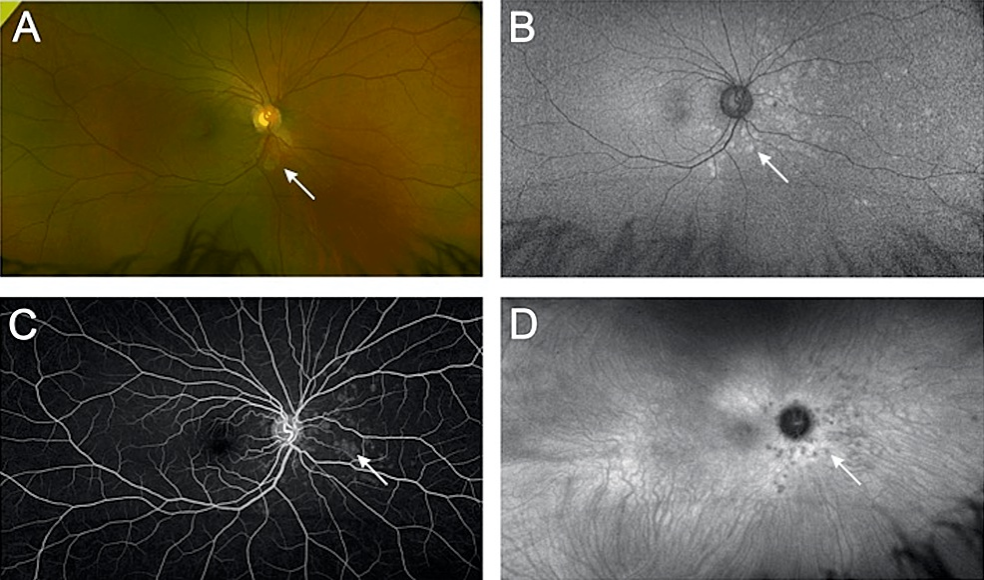
Fundus imaging on presentation of recurrence of MEWDS in the right eye (A) Color photographs revealed multiple white spots nasal to the disk. (B) Fundus autofluorescence (FAF), marked by hyperfluorescent lesions in the nasal to the disk and surrounding the peripapillary region corresponding to the fundus findings. (C) A late-phase intravenous fluorescein angiogram shows spots in the nasal midperiphery. (D) Late-phase indocyanine green angiography revealed numerous hypocyanescent spots nasal to the disk in correspondence to the previously described lesions. MEWDS - multiple evanescent white dot syndrome

Eight months following her initial presentation, the patient presented with flashes in OD. Upon ophthalmic examination, her BCVA was 20/20 OU, no APD was present, and the slit-lamp examination was within normal limits. A right fundus examination revealed multiple white spots nasal to the disk (Figure 2A). The FAF and FA revealed hyperfluorescent lesions nasal to the disk and surrounding the peripapillary region corresponding to the fundus findings (Figure 2B, 2C). The ICG revealed numerous hypocyanescent spots nasal to the disk and corresponding to the previously described lesions (Figure 2D). The patient was diagnosed as experiencing a recurrence of MEWDS in the OD and scheduled for regular follow-up until the condition resolved.

One month following the initial presentation of the symptoms in the OD, the patient presented with a recurrence of flashes. The patient's BCVA remained 20/20 OU. A fundus examination revealed white lesions suggestive of active MEWDS temporal to the macula and brown-colored spots nasal to the disk, suggestive of recovering MEWDS nasally.

**Figure 3:**
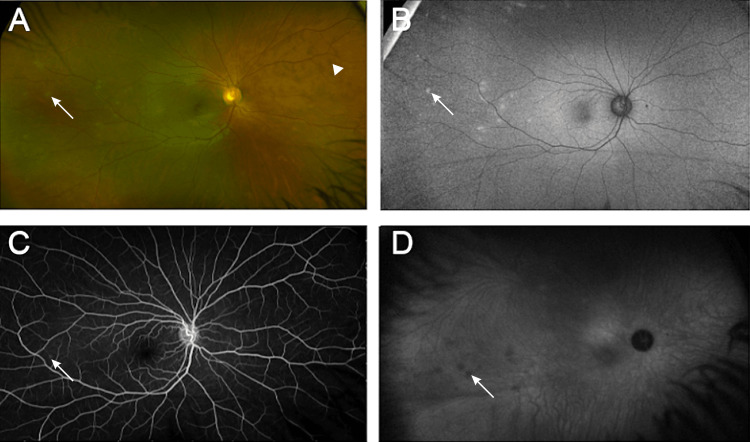
Fundus imaging at the presentation of the second recurrence of multiple evanescent white dot syndrome in the right eye (A) Color photographs reveal white lesions (arrow) temporal to the macula and brown-colored spots (arrowhead) nasal to the disk. (B) Fundus autofluorescence, marked by hyperfluorescent lesions (arrow) in the temporal region corresponding to the fundus findings. (C) A late-phase intravenous fluorescein angiogram shows spots (arrow) in the temporal region. (D) Late-phase indocyanine green angiography reveals numerous hypocyanescent spots (arrow) temporal to the disk and corresponding to the previously described lesions.

The following month, the patient's BCVA remained 20/20 OU. The fundus of the OD was remarkable for brown-colored spots of recovering MEWDS in the temporal region (Figure 3A). An FA was performed and found to be within normal limits. The patient's symptoms resolved, and she received the third dose of the BNT162b2 COVID-19 vaccine 11 months after her initial presentation. On her last follow-up visit, 13 months after presentation, she had a visual acuity of 20/20 OU, and her fundus examination was unremarkable, including the resolution of the multiple relatively ill-defined brown-colored subretinal lesions.

## Discussion

In the literature available about MEWDS, it is suggested that this disease may be triggered by infections or post-vaccination immune reactions [1-3,6,7]. Vaccines may trigger an inflammatory cascade resulting in uveitis by means of molecular mimicry, direct antigen-mediated humoral immune response, or adjuvant-mediated inflammation [3]. The human papilloma, meningococcal, hepatitis A, hepatitis B, COVID-19, influenza, and yellow fever vaccines have been associated with a handful of MEWDS cases [1-3,6,7]. Our patient's encounter with a wide array of antigens incorporated into the meningococcal vaccine, the HPV vaccine, and the three doses of BNT162b2 COVID-19 vaccine, as well as her acquiring of the COVID-19 viral infection, formulated a powerful immune challenge.

Most patients with MEWDS present a single self-limited episode of disease activity, with resolution within a month; nevertheless, our patient presented a total of three episodes, two of which were in the same eye [1-3]. The clinical manifestation of each of our patient's MEWDS episodes involving white dots, photopsia, and blurred vision did not differ from that of classic cases of MEWDS [1-3]. As a result of these characteristics, we believe it was the same disease. She first had MEWDS in her OS 16 days after receiving combined meningococcal and HPV vaccines. Subsequently, she presented two additional episodes of this disease in her OD three months after having received the combination of the second dose of the HPV vaccine and the two doses of BNT162b2 COVID-19 vaccine together with enduring COVID-19 viral infection. To our knowledge, this is the second case of recurrent MEWDS in a patient recently immunized against COVID-19 [3].

Authors have proposed two hypotheses as to why this disease may recur [3]. The first is that if an infectious agent is a cause, then a latent state may be established, which can lead to spontaneous relapses [3]. The second is that if MEWDS represents an autoimmune disorder, repeat exposure to external antigens may precipitate the recurrence of the disease, possibly in the same way numerous autoimmune diseases are characterized by the patient's development of increased immune reactivity [3]. It is likely that until the etiology of this disease is ascertained, the definite cause of these recurrences and how to prevent them will remain uncertain.

**Figure 4:**
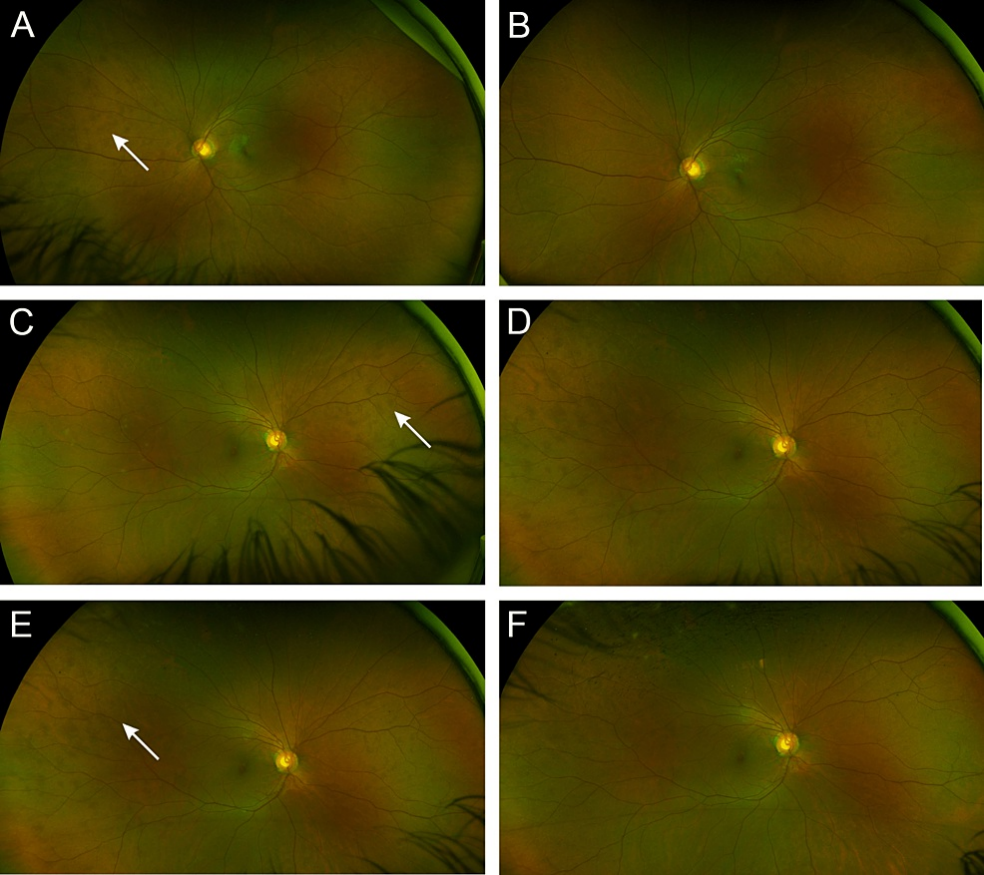
The color fundus photographs of our patient one month after the onset of each MEWDS episode (A) Multiple relatively ill-defined brown-colored subretinal lesions (arrow) are seen in the nasal midperiphery corresponding to the location where the MEWDS lesions were present after the first episode of MEWDS OS. (B) The resolution of brown-colored spots OS in the nasal midperiphery. (C) Brown-colored lesions (arrow) present nasal to the disk after the first recurrence of MEWDS OD. (D) Resolution of brown-colored spots in the right eye in the nasal midperiphery. (E) Brown-colored spots (arrow) are present in the temporal region after the second recurrence of MEWDS OD. (F) The resolution of brown-colored spots OD in the temporal midperiphery. MEWDS - multiple evanescent white dot syndrome

The difficulty in investigating MEWDS lies in the fact that it is characterized by mild and evanescent signs and symptoms [1-3,6,7]. For this reason, the typical whitish dots often described in the posterior pole and the midperiphery may not be present when the patient seeks a consultation [1-3,6,7]. Our patient presented with multiple relatively ill-defined brown-colored subretinal lesions in the later phase of each MEWDS episode she endured (Figure 4). These lesions were possibly the result of an attenuated ellipsoid zone rendering a different color than the retinal pigment epithelium. The brown-colored lesions also corresponded to the spots in the FAF.

## Conclusions

Our case suggests that vaccines may serve as immunological triggers of MEWDS, which further supports the post-infectious or immune-mimicry etiology of this disease. Recurrent MEWDS may occur when an individual is exposed to a powerful immune challenge, such as receiving a wide array of vaccinations in a short period of time. The potential role of COVID-19 vaccines as triggers of recurrent MEWDS should be further studied. To better elucidate such underlying factors as may exist, a thorough vaccination history is of utmost importance when evaluating patients. We believe this case constitutes a previously undescribed finding of ill-defined brown-colored subretinal lesions present in MEWDS, which may prove useful for patients with late presentation of MEWDS.
